# Metal silicide/poly-Si Schottky diodes for uncooled microbolometers

**DOI:** 10.1186/1556-276X-8-177

**Published:** 2013-04-17

**Authors:** Kirill V Chizh, Valery A Chapnin, Victor P Kalinushkin, Vladimir Y Resnik, Mikhail S Storozhevykh, Vladimir A Yuryev

**Affiliations:** 1 A M Prokhorov General Physics Institute of the Russian Academy of Sciences, A M Prokhorov General Physics Institute of the Russian Academy of Sciences, 38 Vavilov Street, Moscow, 119991, Russia; 2 Technopark of GPI RAS, Moscow, 119991, Russia

**Keywords:** Ni silicide, Poly-Si, Schottky diodes, Temperature sensors

## Abstract

Nickel silicide Schottky diodes formed on polycrystalline Si 〈*P*〉 films are proposed as temperature sensors of monolithic uncooled microbolometer infrared focal plane arrays. The structure and composition of nickel silicide/polycrystalline silicon films synthesized in a low-temperature process are examined by means of transmission electron microscopy. The Ni silicide is identified as a multi-phase compound composed of 20% to 40% of Ni_3_Si, 30% to 60% of Ni_2_Si, and 10% to 30% of NiSi with probable minor content of NiSi_2_ at the silicide/poly-Si interface. Rectification ratios of the Schottky diodes vary from about 100 to about 20 for the temperature increasing from 22â„ƒ to 70â„ƒ; they exceed 1,000 at 80 K. A barrier of around 0.95 eV is found to control the photovoltage spectra at room temperature. A set of barriers is observed in photo-electromotive force spectra at 80 K and attributed to the Ni silicide/poly-Si interface. Absolute values of temperature coefficients of voltage and current are found to vary from 0.3%â„ƒ to 0.6%/â„ƒ for forward bias and around 2.5%/â„ƒ for reverse bias of the diodes.

## Background

Recently, outstanding achievements have been made in the development of a novel class of uncooled microbolometer infrared (IR) focal plane arrays (FPAs), the ones based on Si-on-insulator diodes as temperature sensors, whose format has reached 2 megapixels with a noise equivalent temperature difference (NETD) of 60 mK at the frame rate of 15 Hz and the *f*-number of 1; the same group has also demonstrated a VGA FPA with outstanding NETD of 21 mK (at *f*/1, 30 Hz) (see, e. g., [1] and earlier articles cited therein). This success, as well as previous achievements in this field [2-4], stimulates the search for simple complementary metal-oxide semiconductor (CMOS)-compatible technological solutions based on diode bolometers which would be suitable for mass production of IR FPAs with low cost and NETD figures sufficient for many civil applications [5-9]. One of such solutions consists in utilization of metal/poly-Si Schottky barriers for the formation of sets of temperature sensors on bolometer membranes [8,10]. Schottky barrier bolometer arrays seem to be first proposed theoretically for very sensitive cooled bolometers [11]. In this article, nickel silicide Schottky diodes formed on polycrystalline Si 〈*P*〉 films are proposed as thermosensitive elements of monolithic uncooled microbolometer IR FPAs. The possibility of integration of technological process of the silicide-based Schottky diode structure formation into the standard CMOS technology of VLSI manufacturing [12] as well as the possibility of cascade connection of Schottky diodes to increase the temperature sensitivity of bolometer elements of FPA and the use of layers of the diode structures as absorbing coatings in bolometers are advantages of these structures.

## Methods

### Sample preparation and characterization techniques

Schottky barriers were formed on commercial single-crystalline Czochralski-grown silicon wafers (*ρ*=12*Ω*cm, (100), p-type) coated by about 600-nm-thick layer of SiO_2_ formed by thermal oxidation and about 180-nm-thick layer of pyrolytic Si_3_N_4_ (the dielectric layers simulated a design of the supporting membranes of the previously tested bolometer cells [10,13,14]). Films of polycrystalline Si 〈*P*〉 with the thicknesses of about 150 nm were deposited by thermal decomposition of monosilane at the substrate temperature *T*_s_≈620â„ƒ; then they were doped with phosphorus by ion implantation (*E* = 35 keV) to the dose of 5×10^15^ cm ^−2^ and annealed at 700â„ƒ for 30 min. After wafer cleaning in a boiling ammonia-peroxide mixture solution (NH_4_OH/H_2_O_2_/H_2_O = 1:1:4, 10 min) and surface hydrogenation (HF/H_2_O = 1:10, 30 s at room temperature), Ni silicide/poly-Si Schottky diodes were formed by thermal deposition of a nickel film (about 45 nm thick, *T*_s_≈300 K, the residual gas pressure *P*_r_<10^−6^ Torr) from a tungsten crucible followed by annealing at 400â„ƒ in nitrogen for 30 min. Al contacts to poly-Si were formed by thermal deposition from tungsten crucible in vacuum (*P*_r_<10^−6^ Torr, *T*_s_≈300 K) and annealing at 450â„ƒ in nitrogen for 15 min. Aluminum contacts to the top layers of the structures were deposited in the same way but without annealing. Golden wires were welded to the contact pads. Structural perfection and chemical composition of the layers were explored by means of transmission electron microscopy (TEM). Test elements for electrical measurements were formed by contact lithography and had the sizes of about 1 mm. *I-V* characteristics of the Schottky diodes were measured in darkness at different temperatures varied in the range from 20â„ƒ to 70â„ƒ and at the temperature of 80 K. Photovoltage (*U*_emf_) spectra were obtained as described in [15]; for each photon energy (*hν*), the photoresponse value *U*_emf_ was normalized to the number of incident photons. Uncoated satellites were used for the measurement of sheet resistance (*ρ*_s_) of the poly-Si films. The WSxM software [16] was used for TEM image processing.

## Results and discussion

**Figure 1:**
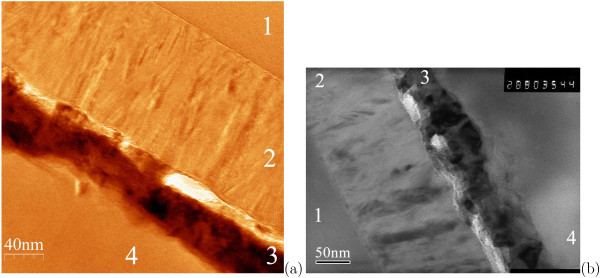
**TEM images demonstrate a Schottky diode film composed of three layers on Si**_**3**_**N**_**4**_**.** (1) is the Si_3_N_4_ substrate film; the diode film consists of (2) poly-Si, (3) nickel silicide, and (4) Al contact layers. (**a**, **b**) Images of different samples with similar structures obtained by the use of different microscopes.

A typical TEM micrograph of the resultant structure (Figure 1) represents images of polycrystalline Ni silicide and polysilicon layers between Si_3_N_4_ and Al films. The Ni silicide film is seen to be composed of a number of phases: at least two phases with the grains close in sizes and comparable volume fractions are distinctly observed by TEM. Bright inclusions are also observed at the Ni silicide/poly-Si interface; we presumably interpret them as residual silicon oxide particles.

It is also seen in Figure 1 that after the formation of the Ni silicide/poly-Si film, the average thicknesses of the Ni silicide and poly-Si layers became 60 and 135 nm, respectively. Using the mass conservation law, this allows us to estimate the density of the silicide film as approximately 7 g/cm^3^ (we adopt the density of poly-Si to be 2.33 g/cm^3^ and the density of the initial poly-Ni film to be 8.9 g/cm^3^). This in turn allows us to roughly evaluate the composition of the silicide layer (the required densities of Ni silicides can be found, e. g., in [17,18]). If we postulate that the silicide film consists of only two phases, as it is stated in [17], then they might be Ni_2_Si and NiSi (the process temperature did not exceed 450â„ƒ and mainly was 400â„ƒ or lower; it is known however that NiSi_2_ - or, according to [19], slightly more nickel-rich compound Ni _1.04_Si _1.93_ - forms at *T*_s_>600â„ƒ (or even >700â„ƒ [18]), whereas NiSi and Ni_2_Si form at *T*_s_>400â„ƒ and 200â„ƒ, respectively [19-21]. According to [17], the appearance of these two low-temperature phases of Ni silicides after annealing in vacuum would be evidence that the original Ni film has been completely (or nearly completely) consumed by the growing Ni_2_Si phase).^a^ In this case, the volume fraction of Ni_2_Si/NiSi ≳ 85:15 (taking into account all uncertainties, the maximum estimate yields 100% of Ni_2_Si); the mass fraction of Ni_2_Si exceeds 88%. This obviously contradicts our TEM observations and makes us assume the presence of the heaviest of the Ni silicides, Ni_3_Si [18], which also may form at low temperatures, especially taking into account the possible presence of oxygen in the metal film that, according to [17,22], impedes diffusion of Ni atoms to Ni/Ni_2_Si interface and, in our opinion, may result in simultaneous formation of Ni_2_Si and Ni_3_Si phases in the silicide film. If our assumption is true, the silicide film might be composed, by a rough estimate, of 20% to 40% of Ni_3_Si, 30% to 60% of Ni_2_Si, and 10% to 30% of NiSi in respective proportions to give a total of 100% of the silicide film volume. The lightest (the least dense) silicide phase having a Si-rich stoichiometry (disilicide) may also be available in the form of a thin diffusion layer at the Ni silicide/poly-Si interface (this does not contradict our observations) [23]; it may affect the barrier height of the whole silicide layer, however [20].

**Figure 2:**
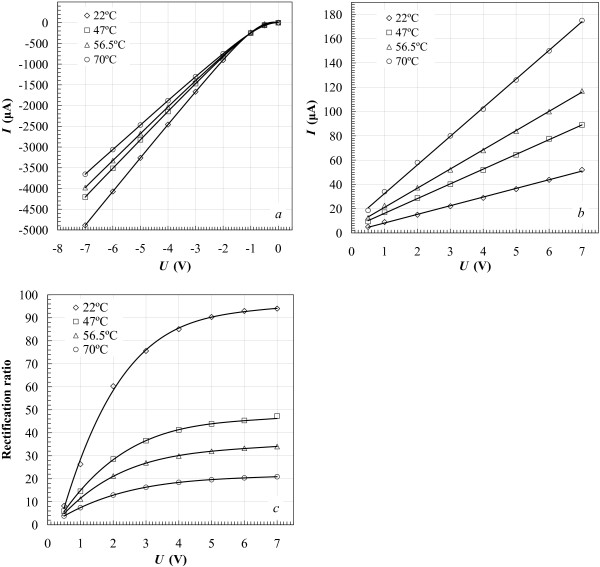
***I*-*V* characteristics of the Ni silicide/poly-Si structure and its rectification ratios at different temperatures.** (**a**) Forward and (**b**) reverse biases; (**c**) rectification ratio vs. the applied voltage.

**Figure 3:**
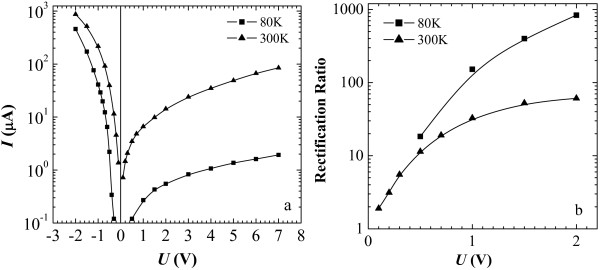
***I*-*V* characteristics of the Ni silicide/poly-Si structure and its rectification ratios at liquid nitrogen and room temperatures.** (**a**) *I*-*V* characteristics and (**b**) rectification ratio as a function of the applied bias.

*I*-*V* characteristics of the structures (Figure 2a,b) with low-resistance poly-Si ($\rho_{s}\approx 270\;\Omega /□$), which forms in our process, manifest a diode behavior with the rectification ratios changing from about 100 to about 20 for the temperature varied from 22°C to 70°C (Figure 2c). At liquid nitrogen temperature, the rectification becomes more pronounced and exceeds 1,000 at biases exceeding 2 V (Figure 3). It should be noticed that at forward bias, the negative lead was set on the silicide top contact pad, whereas the positive one was set on the contact pad to the polysilicon film.

**Figure 4:**
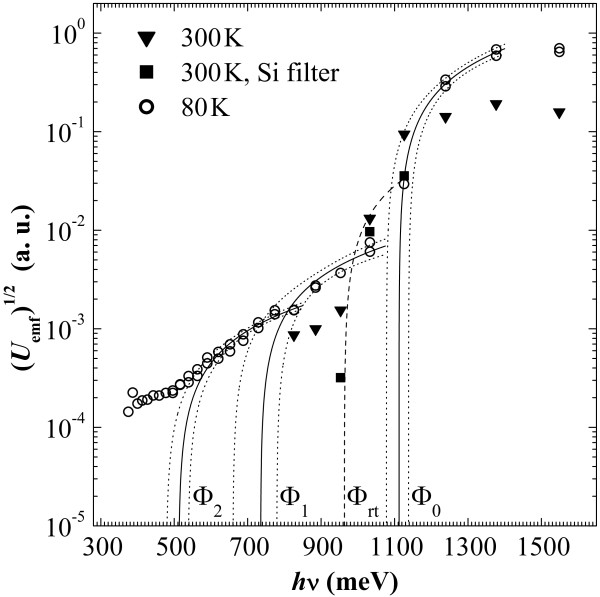
**Photovoltage spectra obtained at room and liquid nitrogen temperatures.***Φ*_rt_ is an estimate of the barrier height derived from the photo-emf spectral measurements at 300 K with and without a Si filter; *Φ*_0_, *Φ*_1_, and *Φ*_2_ are barrier heights estimated from the photo-emf spectral response at 80 K (solid lines show line fits, and dotted ones set 95% confidence bands).

Photo-electromotive force (emf) spectra obtained at 300 and 80 K (Figure 4) demonstrate photoresponse for photons with energies greater and less than the Si bandgap width (*E*_g_) as well as the presence of a number of potential barriers in the diode film. Room temperature measurements with and without a silicon filter have revealed the only barrier with the height *Φ*_rt_≈0.95 eV (note that the negative pole of the photodiode was on the contact pad to the silicide when the diode was illuminated by the white light). A richer collection of barriers has been revealed at 80 K. The highest one nearly coincides in energy with *E*_g_ (*Φ*_0_≈1.1 eV with 95% confidence limits of 1.08 and 1.14 eV). A lower one *Φ*_1_≈0.74 eV (with the 95% confidence limits of 0.66 and 0.78 eV) is close to the values ascribed in the literature to all Ni silicide barriers with n-type Si [17,20,21] (equality of barrier heights of all nickel silicides was explained by the presence of similar diffusion layers in all nickel silicide/silicon interfaces [20]). Estimation of the lowest one yields a figure of *Φ*_2_≈0.51 eV (the 95% confidence band is from 0.48 to 0.54 eV); a barrier of this height, to our knowledge, has never been connected with a Ni silicide/Si transition in the literature.^b^ However, we attribute all the above barriers to the Ni silicide/poly-Si interface. Our reasoning is as follows. The band structure of a polysilicon film is known to be spatially inhomogeneous: A strong potential relief is associated with grain boundaries [24]. In n-Si, even in the heavily doped n ^+^ one, there may exist depleted or even p-type spatial domains [24] which, on the one hand, as a result of band-to-band transitions, may be sources of electron-hole pairs. In turn, these pairs are separated by the potential relief and generate the photo-emf of the observed polarity because, despite that the potential peaks should be more or less symmetrical and the electron-hole pairs should arise with close likelihoods on both their slopes, a part of electrons escapes from the Si film accumulating in silicide, whereas holes are localized at the grain boundaries. This process may give rise to the photovoltage under irradiation by photons with energies $\mathrm{h\nu}≳E_{g}$. In addition to charge separation on opposite sides of the film, this process also increases the potential relief. On the other hand, grain boundaries may serve as potential barriers for electrons localized in n ^+^-Si grains segregating them from the Ni silicide film and producing the photo-emf of the observed polarity due to electron injection into the silicide under the effect of photons with *hν*<*E*_g_; n ^+^-Si potential valleys adjoining the Ni silicide film form ohmic contacts. This argumentation explains the presence of the only barrier *Φ*_rt_ detected at room temperature as well as the observed polarity of both the resultant photovoltage and the forward current.

**Figure 5:**
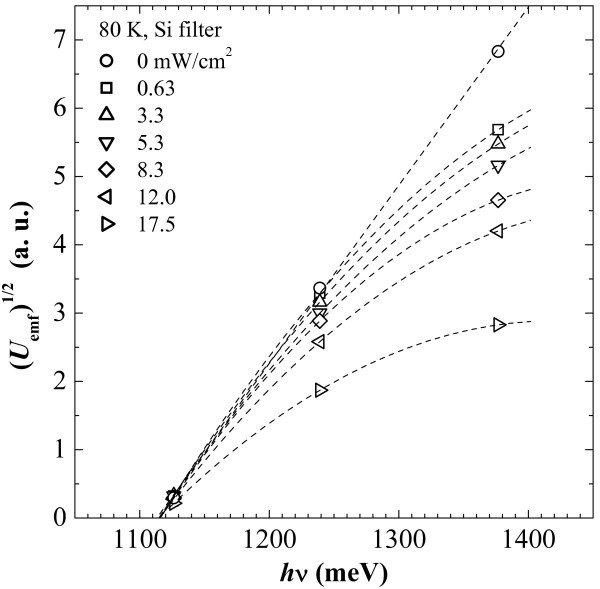
**Photovoltage spectra obtained at 80 K.** The diode is irradiated by the light of a tungsten lamp through a Si filter. The power density of light with *hν*<*E*_g_(300 K) on the diode is shown in the legend in mW/cm^2^; dashed lines are guides to the eye.

A model of processes taking place at liquid nitrogen temperature is some more tricky. As it follows from the *I*-*V* characteristics (Figure 3), the free electrons are partially frozen out in the structure and the Fermi level moves down that increases the barriers for electron photoinjection into the silicide film. It makes *Φ*_rt_ move to the right in energy to appear in the photovoltage spectra as *Φ*_0_. Two processes can be mixed in this conditions, band-to-band transition with separation of electron-hole pairs and electron injection into the silicide over the potential barrier, both generating photo-emf. In addition, a reduction of *n* may increase barriers at the interface [25,26]; a usual Ni silicide barrier (around 0.7 eV) may be completely restored at some domains or be still reduced (around 0.5 eV) at different places. Hole injection into the silicide layer from polysilicon grain boundaries may become more probable over reduced barriers to holes. This statement finds confirmation in the spectra plotted in Figure 5 which have been obtained under irradiation of a diode by a wide-band IR radiation of a tungsten bulb filtered by a polished Si wafer (*hν*<*E*_g_(300 K)). It is seen in the spectra that the higher the power density of the incident radiation on the sample, the stronger the curves bow in the high-energy part of the graph and the lesser values of the photo-emf are detected. It may be caused by injection of holes from potential wells at grain boundaries of poly-Si into the silicide film because of additional wide-band IR lighting of the sample resulting in charge reduction of both the silicide and polysilicon layers.

Thus, a set of competing processes becomes possible at 80 K. Non-uniformity of the spatial potential throughout the Ni silicide/poly-Si interface may locally act in favor of one of these competing processes. As a consequence, the impact of several barriers is observed in the photoresponse spectra in the order of magnitude of contribution of processes associated with them to the resultant photo-emf in different spectral ranges.

**Figure 6:**
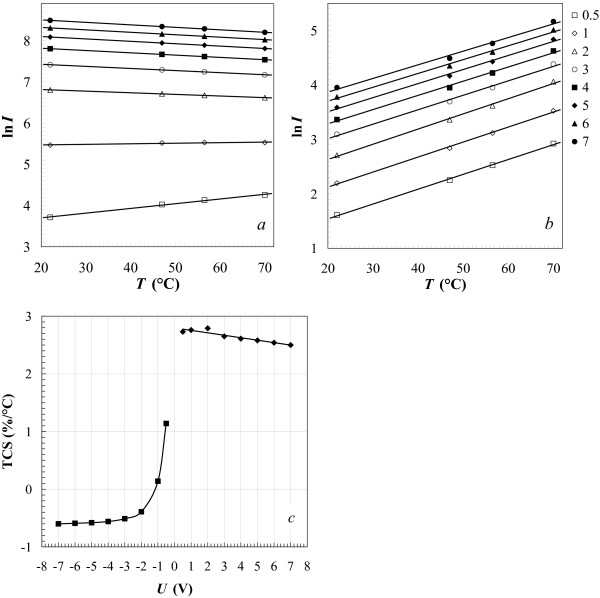
**Temperature dependences of current and temperature coefficient of signal.** Temperature dependences of current are presented for fixed voltages on a Ni silicide/poly-Si Schottky diode and temperature coefficient of signal (current) is plotted for each branch of the *I-V* characteristics. (**a**) Forward and (**b**) reverse currents (the legend represents the applied bias in volts for each line). (**c**) Temperature coefficient of current vs. fixed voltage on the structure; negative and positive values of *U* in (c) correspond to forward and reverse biases, respectively.

**Figure 7:**
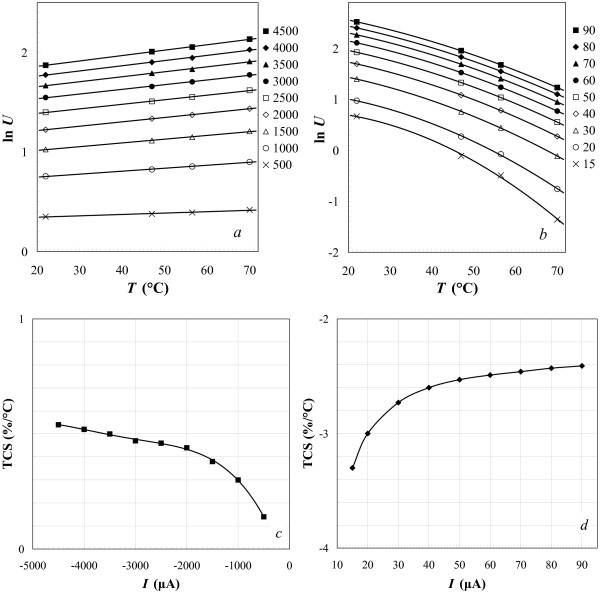
**Temperature dependences of voltage and temperature coefficient of signal.** Temperature dependences of voltage are presented for fixed currents through a Ni silicide/poly-Si Schottky diode and temperature coefficient of signal (voltage) is plotted for each branch of *I-V* characteristics. (**a**) Forward and (**b**) reverse biases (the legends represent the currents in μA for each line). (**c**, **d**) Temperature coefficient of voltage for each branch of *I-V* characteristics vs. fixed current through the structure. To derive the graph (d), the curves in (b) were linearized in the interval from 20â„ƒ to 60â„ƒ. Negative and positive values of *I* in (c) and (d) correspond to forward and reverse biases, respectively.

Investigating the temperature dependences of the *I*-*V* characteristics close and above the room temperature, we have found the thermal sensitivity of the diodes to be sufficiently high to consider them as potential elements of uncooled bolometers. Figure 6a,b demonstrates temperature dependences of the forward and reverse currents of the diodes (*I*), respectively, for fixed (and stabilized) voltages (*U*). Temperature coefficient of the sensor current TCS =*d*[ ln*S*(*T*)]/*dT*, where *S*=*I*, derived from the graphs presented in Figure 6a,b as a function of bias voltage (Figure 6c) varies from −0.3%/â„ƒ to −0.6%/â„ƒ for the forward bias and remains nearly constant around 2.5 %/â„ƒ for the reverse bias. Notice that at small values of the forward bias, TCS is positive but rapidly drops with the growth of the absolute bias and equals 0 at *U*≈−1 V. We think that the negative TCS may result from the metallic behavior of the poly-Si film as a function of temperature. Temperature dependences of the voltage drop across the diode *U* for fixed (and stabilized) forward and reverse currents *I* are shown in Figure 7a,b. The temperature coefficient of voltage TCS (*S*=*U*) derived from the graphs depicted in Figure 7a,b (the curves in panel (b) are linearized over an interval from 20â„ƒ to 60â„ƒ) varies from 0.3%/â„ƒ to 0.6%/â„ƒ for forward bias and from −3%/â„ƒ to −2.4%/â„ƒ for reverse bias (Figure 7c,d).

As of now, we foresee two ways of improvement of electrical properties of the structure. The first of them consists in modification of the Schottky barrier formation process proposed in [27] which enables production of poly-Si/Ni polycide Schottky diodes with rectification ratios as high as 10^6^. The other possibility is to replace poly-Si by *α*-Si:H and to apply the metal-induced crystallization to form diodes nearly as perfect as those produced on the basis of single-crystalline Si [8,28-30]. Each of these alternatives in principle could enable the development of high-performance monolithic Schottky diode microbolometer IR FPAs.^c^

## Conclusion

In summary, nickel silicide Schottky diodes formed on polycrystalline Si 〈*P*〉 films are proposed as temperature sensors of monolithic uncooled microbolometer IR focal plane arrays. The structure and chemical composition of the Schottky diodes have been examined by TEM. The Ni silicide has been identified as a multi-phase mixture composed of 20% to 40% of Ni_3_Si, 30% to 60% of Ni_2_Si, and 10% to 30% of NiSi with probable minor content of NiSi_2_ at the silicide/poly-Si interface. *I*-*V* characteristics of the diodes studied at different temperatures demonstrate the rectification ratios varying from about 20 to about 100 when the temperature changes from 70â„ƒ to 22â„ƒ and exceeding 1,000 at 80 K. A barrier of around 0.95 eV has been found to control the photovoltage spectra at room temperature. Three barriers with approximate heights from 1.08 to 1.14 eV, from 0.66 to 0.78, and from 0.48 to 0.54 eV have been observed in photo-emf spectra at 80 K and associated with the Ni silicide/poly-Si interface. Absolute values of temperature coefficients of voltage and current have been found to vary from 0.3%/â„ƒ to 0.6%/â„ƒ for the forward biased structures and around 2.5 %/â„ƒ for the reverse biased ones.

## Endnotes

^a^We cannot discriminate between *δ* and *θ* phases of Ni_2_Si [18] and, following [17], suppose that only the *δ* phase is present; the experimental value of its density, taken from [18], makes 7.23 g/cm^3^, whereas its X-ray density (7.405 g/cm^3^) coincides in various sources [17,18].^b^A barrier of this height is attributed to the Ni/Si interface in [21], yet we have not observed a direct contact of Ni to Si by TEM after the silicide film formation.^c^Notice also that there is an additional advantage of the considered structures with Schottky barriers. They may be applied both as temperature sensors of bolometers for the detection in mid-IR or far-IR and as photonic sensors for the detection in near-IR and visible spectral ranges.

## Abbreviations

CMOS: Complementary metal-oxide semiconductor; emf: electromotive force; FPA: Focal plane array; IR: Infrared; NETD: Noise equivalent temperature difference; TCS: temperature coefficient of the sensor signal.

## Competing interests

The authors declare that they have no competing interests.

## Authors’ contributions

KVC participated in the design of the study, carried out the experiments, performed data analysis, and participated in the discussions and interpretation of the results. VAC participated in the design of the study and took part in the discussions and interpretation of the results; he also supervised the research performed by young scientists and students. VPK participated in the design of the study and took part in the discussions and interpretation of the results. VYR performed the TEM studies and took part in the discussions and interpretation of the results. MSS investigated the photo-emf spectra; he carried out the experiments, performed data analysis, and took part in the discussions and interpretation of the results. VAY conceived and designed the study, performed data analysis, and took part in the discussions and interpretation of the results; he also supervised the research project. All authors read and approved the final manuscript.

## Authors’ information

KVC is a junior research fellow, VAC is a leading research fellow, and MSS is a PhD student at the Laboratory of Nanophotonics, Department of Applied Thermography, Prokhorov General Physics Institute, Russian Academy of Sciences. VYR is a senior research fellow and VPK is the head of the Laboratory of Medium IR-range Crystalline Lasers at the Department of Applied Thermography, Prokhorov General Physics Institute. VPK is also a co-founder and a board member of Technopark of GPI RAS and a co-founder and a partner of Thermographic Systems Ltd. VAY is the head of the Department of Applied Thermography and the Laboratory of Nanophotonics at Prokhorov General Physics Institute; he is also a co-founder and a board member of Technopark of GPI RAS and a co-founder and a partner of Thermographic Systems Ltd.
